# miR-182 aids in receptive endometrium development in dairy goats by down-regulating PTN expression

**DOI:** 10.1371/journal.pone.0179783

**Published:** 2017-07-05

**Authors:** Lei Zhang, Xiaorui Liu, Junze Liu, Zhanqin Zhou, Yuxuan Song, Binyun Cao, Xiaopeng An

**Affiliations:** College of Animal Science and Technology, Northwest Agriculture and Forestry University, Yangling, Shaanxi, China; Baylor College of Medicine, UNITED STATES

## Abstract

Increasing evidence has shown that miRNAs play important roles in endometrium development during the menstrual cycle in humans and many other animals. Our previous data indicated that miR-182 levels increase 15.55-fold and pleiotrophin (PTN) levels decrease 20.97-fold in the receptive endometrium (RE, D15) compared with the pre-receptive endometrium (PE, D5) in dairy goats. The present study shows that miR-182 is widely expressed in different tissues of dairy goats and that its expression levels are regulated by E2 and P4 in endometrial epithelium cells (EECs). We confirmed that PTN is a target of miR-182 and that miR-182 regulates the protein levels of AKT, Bcl-2, FAS, MAPK, Caspase-3 and SP1 in EECs. Furthermore, miR-182 up-regulates or maintains the expression levels of osteopontin (OPN), cyclooxygenase-2 (COX-2) and prolactin receptor (PRLR) in EECs, suggesting that miR-182 is an important regulatory factor in the construction of endometrial receptivity in dairy goats. In conclusion, miR-182 participates in the development of endometrial receptivity by down-regulating PTN and affecting the expression of select apoptosis-related genes and increasing or maintaining the expression levels of OPN, COX-2 and PRLR in the EECs of dairy goats.

## Introduction

The endometrium is lined by epithelial cells (EEC) and is supported by a stromal cell (ESC) foundation [1]. The EEC layer is essential for reproductive success, including nutrition supply for embryo implantation and pregnancy maintenance [2, 3]. Endometrial receptivity is the result of both the synchronized and integrated interaction among many factors, and the reconstruction of the endometrium that allows for embryo adhesion [4]. Timing of implantation and endometrial receptivity have major impact on the establishment of a successful pregnancy and on the future health of offspring [5]. Although many factors participate in the development and progression of endometrial receptivity, the underlying molecular mechanisms are not very clear. Therefore, a better understanding of the development of endometrial receptivity, reproductive function and fecundity of animals is urgent.

In recent years, many miRNAs had been identified in the endometria of human [6–8] and mice [9]. Further studies reported that miR-30b, miR-30d, and -miR-494 are differentially expressed in pre-receptive and receptive endometria in human [10], suggesting that miRNAs played important roles in gene reprogramming during the formation of endometrial receptivity. In addition, miR-145 suppresses embryo-epithelial juxtacrine communication in the procession of embryo attachment by modulating the expression levels of IGF1R in endometria during WOI [11]. Together with its family members (miR-96 and miR-183), miR-182 was described in mouse neurosensory cells, specifically in the retina, inner ear, and dorsal root ganglia [12–14]. Several studies have reported that miR-182 up-regulated in various cancer types, including prostate cancer [15, 16], glioblastoma [12], hepatocellular carcinoma [17]. What’s more, miR-182 could promote cellular differentiation by regulating the expression of snail family transcriptional repressor 2 (SNAI2) and induce the mesenchymal-to-epithelial transition [18]. The present study shows that miR-182 is widely expressed in different tissues in dairy goats, and the levels vary in association with the concentrations of E2 and P4 in EECs. To explore the role of miR-182 in EECs, miR-182 mimics and inhibitors were used to perform miR-182 overexpression or knockdown, respectively. Furthermore, EECs treated with a miR-182 inhibitors became round, the microvilli on the surface of the cell membrane disappeared, and the cell membrane sprouted and formed apoptotic bodies. Apoptosis, or the highly orchestrated form of programmed cell death in which cells neatly commit suicide without triggering an inflammatory response in the tissue, is becoming a relevant event in the study of reproductive physiology [19]. Thus, cell proliferation, cell cycle and apoptosis events of EECs were also analyzed after treatment with a miR-182 mimics or inhibitors.

PTN (also called HBGF8), an 18-kDa heparin-binding growth factor, shares 50% sequence homology with midkine [20–23]. It is a secreted cytokines [24] that plays roles in diverse biology process, such as cell adhesion, migration, survival, growth, and differentiation [20, 22, 25]. Further study showed abnormalities on reproduction and development were observed in the PTN-KO mice [26]. What’s more, PTN was stimulated by pregnancy, displayed a higher expression in the caruncular (C) areas over the intercaruncular (IC) areas in bovine [27], and it increased in murine implantation sites during decidualization [28]. Considering the fact that PTN mainly expressed in the C areas of bovine endometrium, it may participate in the proliferation of stroma cells, and in the decidualization-like process [29] in dairy goats.

Our previous sequencing data indicated that miR-182 expression was increased 15.55-fold in the receptive endometrium (RE, D15) compared with the pre-receptive endometrium (PE, D5) in dairy goats [30], and its predicted target gene, PTN, was down-regulated 20.97-fold [31]. Thus, we inferred that miR-182 may participate in regulating dynamic changes in goat uterine gene expression patterns via down-regulated PTN. After confirming the increase of miR-182 and the low abundance of PTN mRNA in the RE of healthy multiparous dairy goats, the psiCHECKTM-2 reporter plasmid, RT-qPCR, and Western blotting (WB) were used to confirm that miR-182 down-regulated the expression of PTN in EECs of dairy goats. Furthermore, the protein levels of Bcl-2, FAS, MAPK, Caspase-3, SP1, and other marker genes of endometrial receptivity (OPN, VEGF, COX-2, PRLR) were analyzed in EECs that were treated with a miR-182 mimics or inhibitors.

## Materials and methods

### Ethics statement

All animals in this study were maintained according to the No. 5 proclamation of the Ministry of Agriculture, P. R. China. And animal protocols were approved by the Review Committee for the Use of Animal Subjects of Northwest A&F University.

Goats were euthanized when the goats lost consciousness caused by intravenous injection of barbiturate (30mg/kg). Endometrium samples from 5 goats at gestational day 5 and 5 goats at gestational day 15 were obtained from the anterior wall of the uterine cavity [30, 32]. Tissues were fixed in 10% formaldehyde in phosphate-buffered saline (PBS) for immunohistochemical studies; tissues were fixed in 2.5% Glutaraldehyde for Scanning Electron Microscopy (SEM); Another tissues were fixed in 4% paraformaldehyde for Immunohistochemistry (IHC).

### Primary cell cultures

Primary dairy goat endometrial epithelial cells (EECs) were isolated and purified with the methods of trypsin enzymic digestion, differential centrifugation and differential attachment, and then the cells were observed and identified by light microscope [1, 33]. Briefly, immediately after the goats were euthanized, the uterus was placed into PBS supplemented with penicillin (100 IU/mL) and streptomycin (50 mg/mL), and then was transported to the laboratory within 2 h. The tissue was first washed with 75% alcohol for 1 min and then washed three times with PBS to eliminate alcohol. Endometrium was harvested from the uterus by sterile scalpel under sterile conditions, and then was incubated at 37°C in cell culture dish with 5-mL 0.25% trypsin for approximately 2 h, fetal bovine serum (FBS) was added to terminate digestion. The mucus and undigested organization were removed from cells suspension by 150 μm screen, the EECs were centrifuged (500× g, 10 min), resuspended in DMEM/F12 containing 10% FBS (HyClone, Logan, UT, USA). To isolate stromal cells, the supernatant fluid was centrifuged at 1000× g for 10 min, and then the stromal cell-rich fraction (supernatant fluid) was removed.

### RNA extraction and RT-qPCR

Total RNA was extracted using Trizol reagent (TaKaRa, Dalian, China) following the manufacturer′s instructions. RT-qPCR was performed using the Bio-Rad CFX 96 Real Time Detection System and SYBR Green PCRMasterMix (TaKaRa, Dalian, China) in a 20 μl reaction according to the manufacturer′s instruction. All primers for the RT-qPCR are shown in Table 1, and the PCR products further identified by Sanger sequencing. The relative expression levels were calculated using the equation N = 2^-ΔΔCt^.

**Table 1:** The RT-qPCR Primers used in the present study.

gene	GenBank accession No.		Primer sequences (5′→3′)
**GADPH**		AF_030943.1	F: GCAAGTTCCACGGCACAG
			R: GGTTCACGCCCATCACAA
**PTN**		XM_005679509.1	F: TCTCCATTTCCCTTCCTTCC R: TCTCTCTCCACTTCGGCTTT
**PTN**		XM_005679509.1	F: GCCTCGAGGACCGTGAAAAGGACATC R: GCGCGGCCGCCAGCATCACCTTGATTTA
**18S**		/	F: GTGGTGTTGAGGAAAGCAGACA R: TGATCACACGTTCCACCTCATC
**U6**		/	F: CTCGCTTCGGCAGCACA R: AACGCTTCACGAATTTGCGT
**miR-182-Loop**		/	gtcgtatccagtgcagggtccgaggtattcgcactggatacgacAGTGTGAG
**miR-182-FW**		/	ggTGAAAAGTTCGTTCGG
**Reverse Primer**		/	GTGCAGGGTCCGAGGT

Note: the underscore characters were restriction enzyme cutting site of xho І and not І.

### Cell transfection

A mature miR-182 mimics (5’-TTTGGCAATGGTAGAACTCACACT-3’) and inhibitors (5’-AGUGUGAGUUCUACCAUUGCCAAA-3’), a nonspecific control (NC) and NC-inhibitors were synthesized by GenePharma (Shanghai, China). EEC cells were plated at a density of 7.5×10^5^ in 6-well plates, seventy percent confluent cells were transfected with miR-182, miR-182 inhibitors, NC, and NC-inhibitors at final concentrations of 100 nM using the X-tremeGENE siRNA Transfection Reagent (Roche, Switzerland) according to the manufacturer′s protocols. Stem-loop qPCR was used to detect the transfection efficiency of miR-182 in this study [34].

### Analysis of cell proliferation, cycle and apoptosis

The methyl thiazolyl tetrazolium (MTT) (Sigma, St. Louis, MO, USA) colorimetric assay was used to evaluate the proliferation of EECs. Briefly, the cells were seeded in 96-well plates at a density of 2×10^3^ cells/well; the cells were transfected with miR-182, miR-182 inhibitors, NC, and NC inhibitors, and then were incubated at 37°C in a 5% CO_2_ incubator for 24 h after the cells adhered to the plate; and then the MTT reagent (0.5 mg/mL) were added into 96-well plates and incubated for 4h. The relative number of viable cells, which is directly proportional to the production of formazan crystals solubilized by DMSO, the absorbance was measured at a wavelength of 490 nm as previously described [35].

To evaluate the mechanism of the cell growth deficiency in miR-182-transfected EEC, cell cycle staining Kit was used (Liankebio, Hangzhou, China) according to the manufacturer′s instruction. Cells were harvested 48 h later and washed three times by cold PBS, and then fixed in 70% ethanol in PBS at -20°C overnight. And then the cells were incubated with 0.5 ml of propidium iodide (PI) staining buffer, which contains 200 μg/ml RNase A and 50 μg/ml PI, at 37°C in the dark for 30 min [36]. Analyses were performed on BD LSR flow cytometer (BD Biosciences, San Diego, CA).

Cell apoptosis analysis was carried out using Annexin V-FITC/PI apoptosis kit [37] (Liankebio, Hangzhou, China) according to the manufacturer′s instructions. After being treated with miR-182 mimics or miR-182 inhibitors for 48 h, EEC cells were collected and incubated with Annexin V-FITC and PI at room temperature for 5 min in the dark. After adding 300 ml PBS to each sample, and cell apoptosis was detected with flow cytometer (BD Biosciences, San Diego, CA). Annexin-V-positive and PI-negative cells were defined as early apoptotic cells, and the late apoptotic cells were Annexin-V and PI positive cells.

### Scanning Electron Microscopy (SEM)

The effect of miR-182 on the surface morphology of EEC was detected by SEM (Scanning Electron Microscopy) [38]. In short, glutaraldehyde-fixed (2.5%) samples were thoroughly washed with PBS buffer, dehydrated in graded ethanol, placed in 2% isoamyl alcohol for 3 hours, and underwent critical point drying. The samples were attached to the sample stage for observation with the surface (endometrial cavity surface) up and painted with silver conductive plastic using a vacuum coating apparatus for coating metal samples. Then, the samples were observed under the JSM-6330F SEM (JEOL, Japan).

### Immunohistochemistry (IHC)

The paraformaldehyde-fixed (4%) samples were embedded with paraffin, and sectioned. And then the sections were deparaffinized in xylene and rehydrated followed by antigen retrieval in sodium citrate buffer for 10 min in a microwave oven at 100°C. Endogenous peroxidase was inhibited by incubation with 3% hydrogen peroxide for 10 min at RT, and then the sections were blocked in 10% normal goat serum for 30 min and incubated with a primary antibody (as be showed in Table 2) at 4°C overnight.

**Table 2:** The antibody used in the present study.

Name	Manufacturer		Product Number	Species of the antigen	Species of the antibody	method for purification
**β-Actin**		Beyotime, Shanghai, China	AA128	Human	Mouse	AP
**PTN**		Boster Co, Wuhan, China	BA1369-2	Mouse	Rabbit	AP
**AKT**		Beyotime, Shanghai, China	AA326	Mouse	Rabbit	AP
**p-AKT**		Beyotime, Shanghai, China	AA331	Mouse	Rabbit	AP
**Bcl-2**		Beyotime, Shanghai, China	AB112	Human	Rabbit	AP
**FAS**		Abcam, Cambridge, U.K.	ab82419	Mouse	Rabbit	AP
**MAPK (p44/42)**		Beyotime, Shanghai, China	AM076	Rat	Rabbit	AP
**p-MAPK (p44/42)**		Beyotime, Shanghai, China	AM071	Human	Mouse	AP
**Caspase-3 (active form)**		Beyotime, Shanghai, China	AC033	Human	Rabbit	AP
**Sp1**		Boster Co, Wuhan, China	BA1402	Human	Rabbit	AP
**OPN**		Boster Co, Wuhan, China	BM0032	Human	Mouse	AP
**VEGF**		Boster Co, Wuhan, China	BA0407	Human	Rabbit	AP
**LIF**		Boster Co, Wuhan, China	BA1239-2	Rat	Rabbit	AP
**PRLR**		Boster Co, Wuhan, China	BA3818	Human	Rabbit	AP

Note: AP, affinity purification.

The tissue sections were incubated with a biotinylated secondary antibody for 30 min at 37°C. After washing for 10 min in TBS, the colour reaction was developed with the substrate diaminobenzidine (DAB; Zytomed Systems, Berlin, Germany) according to the manufacturer’s instructions. Finally the sections were washed under running tap water for 5 min, counterstained to haematoxylin (VWR, Carnaxide, Oeiras, Portugal). The relative density of the positive cells (density/area) in each slide was analyzed using Image-Pro Plus 6.0 Software (Media Cybernetics, USA)[39].

#### 3′-UTR constructs/luciferase assay

We screened target genes for miR-182 using microRNA.org (http://www.microrna.org/). And the results showed that there was a target site for miR-182 in the 3′UTR PTN of mRNA. To generate reporter constructs for luciferase assays, the full-length 3′UTR of PTN were cloned and inserted downstream of the luciferase gene in the psiCHECKTM-2 vector (Promega, USA), and the mutated plasmid was constructed by inserting PTN 3′ UTR with mutated miR-182 binding site between the XhoI and NotI sites immediately downstream of the Renilla luciferase gene. The wild-type (psiCHECK-PTN-WT) or mutated (psiCHECK- PTN-UTR-Mut) plasmid was co-transfected with the miR-182 mimics or inhibitors into 293T cells.

HEK293T cells were plated in 24-well plates at 70% confluency. The adherent cells were cotransfected with 0.5 μg of luciferase reporter and miR-182 mimics, miR-182 inhibitors, NC, or NC inhibitors (100 nM). At 24h posttransfection, firefly (hluc+) and Renilla (hRluc) luciferase activities were measured with the Dual-Glo luciferase assay system according to the manufacturer′s instructions (Promega, USA) by a thermo scientific varioskan flash (Thermo scientific, USA), the hluc+ gene was used as reference gene to correct the variation of transfection efficiency, the relative luciferase activity was calculated as hRluc/hluc+. Experiments were performed three times in triplicate.

### Protein extraction and Western blot analysis

Protein extraction was performed as described previously [40]. Briefly, cells were harvested and lysed in RIPA buffer (Roche Applied Science, Indianapolis, IN, USA). After 30 minutes′ standing at 4°C, centrifuged (11 000×g) for 10 min. Soluble protein in the supernatants was collected and total protein concentration was determined using the Bio-Rad DC Protein Assay, and then diluted with gel loading buffer (Beyotime, Shanghai, China) and boiled for 8 min.

Thirty micrograms of protein from each treatment was subjected to 12% SDS-polyacrylamide gel electrophoresis and transferred onto nitrocellulose membranes (Millipore, Bedford, MA, USA) at 100 V for 1.5 h in ice bath. Non-specific binding sites were blocked with 5% fat-free powdered milk (blocking solution) TBS-buffered saline plus Tween 20 [0.2% (vol/vol)] (TBST) at room temperature for 2 h. After three washes for 10 min each with TBST, membranes were incubated with primary antibodies (Table 2) overnight at 4°C. After this incubation, membranes were washed three times and then incubated at room temperature for 2 h with the horseradish peroxidases (HRP)-conjugated second antibody at 1:1000 dilutions. After three washes for 5 min each with TBST, proteins were detected using enhanced chemiluminescence (Advansta, California, USA). Quantification was performed using the Quantity One program (Bio-Rad, California, USA).

### Statistical analysis

All the data were processed with SPSS 17.0 (SPSS Inc., Chicago, IL, USA). One-way ANOVA was used to compare the differences, and the method of the least significant difference (LSD) was used for further analysis, and the differences were considered significant when P was <0.05 and very significant when P was <0.01.

## Result

### The expression of miR-182 in dairy goats

**Fig 1:**
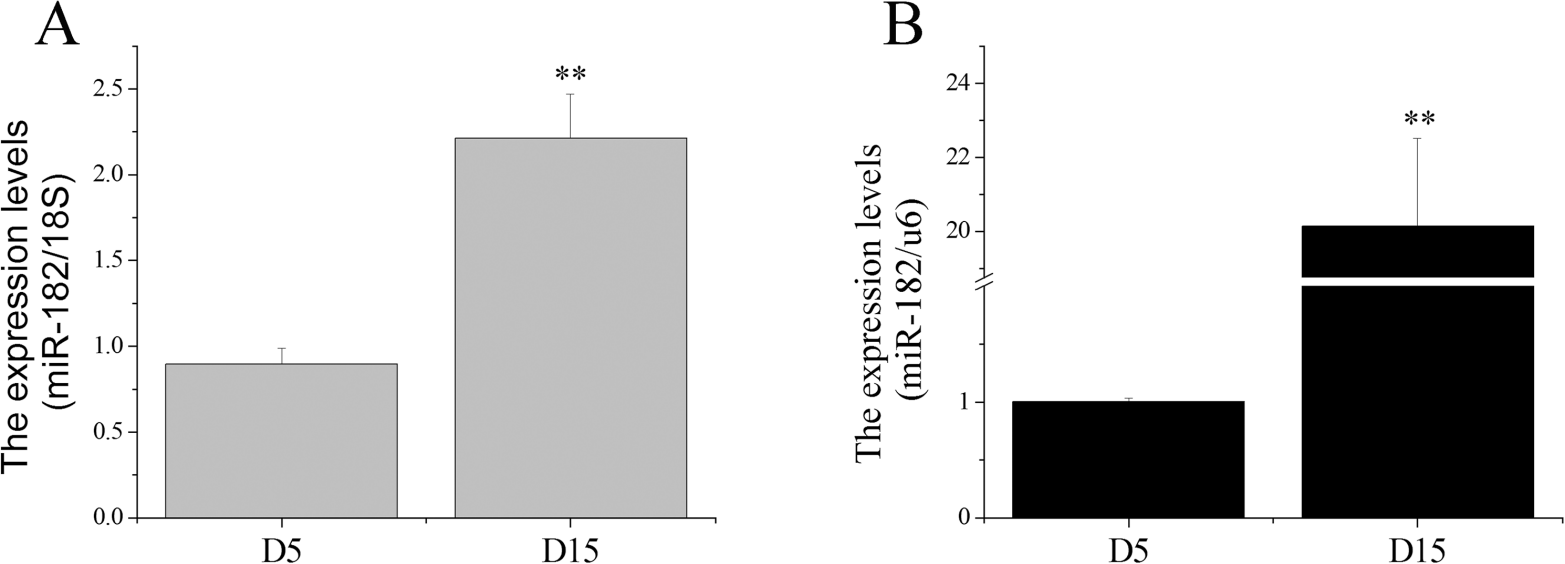
Expression levels of miR-182 in endometrium at D5 and D15. The miR-182 levels in endometrium at D5 and D15 were detected by stem-loop qRT-PCR, 18S rRNA (A) and U6 (B) were used as the references. The values were showed as “means ± SD” (n = 5×3), ** indicates that P < 0.01.

Our previous study indicated that miR-182 was a differentially expressed miRNA and had a 15.55-fold increase in the receptive endometrium (RE) compared with the pre-receptive endometrium (PE) in dairy goats [30]. Therefore, we inferred that miR-182 may participate in regulating dynamic changes in goat uterine gene expression patterns that occur during the transition from the pre-receptive to the receptive phase. Total RNAs were extracted and stem-loop qRT-PCR was used to validate the expression levels of miR-182 compared with the 18S rRNA and U6 reference controls in the dairy goat endometrium. The results showed that miR-182 expression level in the RE was higher (Fig 1) than that in the PE using either reference, which was inconsistent with previous sequencing data.

**Fig 2:**
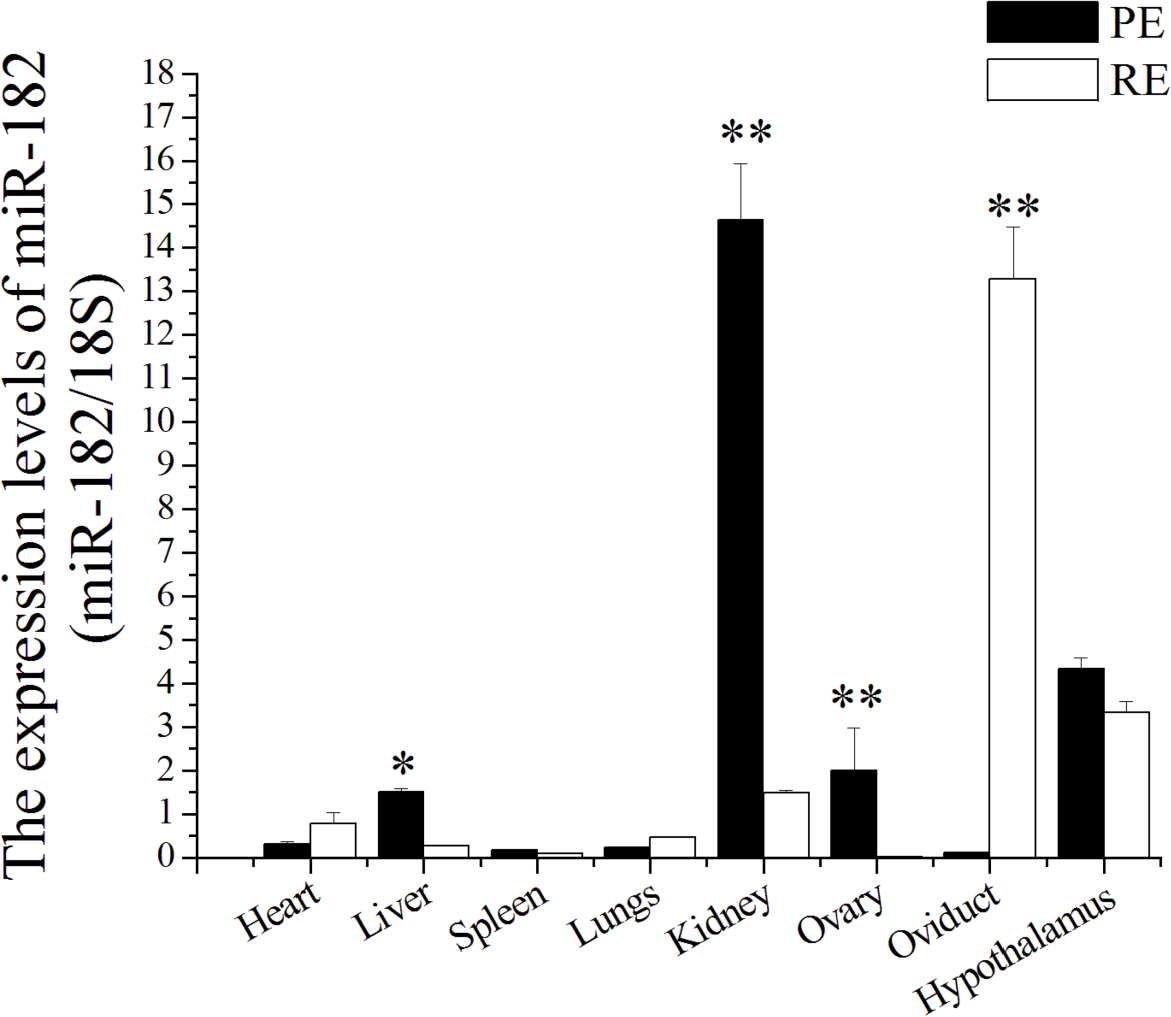
miR-182 was expressed in various tissues of dairy goats. The miR-182 levels in different tissues were measured by Stem-loop RT-qPCR and normalized to 18S. Values were showed as “means ± SD” in receptive endometrium (RE, D15) that were relative to pre-receptive endometrium (PE, D5); n = 5×3. ** indicates that P < 0.01 and * indicates that P < 0.05.

Furthermore, the expression levels of miR-182 in various tissues of dairy goats were studied, and miR-182 was found to be widely expressed in different tissues at both biophysical stages. The highest expression levels were observed in the kidney, followed (in order) by the hypothalamus, ovary, liver, lung, spleen, and oviduct at gestational day 5 (D5, PE), whereas at gestational day 15 (D15, RE), the highest levels were observed in the oviduct, followed (in order) by the hypothalamus, kidney, heart, lung, liver, spleen, and ovary (Fig 2). The liver showed the largest difference in miR-182 levels between D5 and D15; the level decreased 120.63-fold at D15 compared with that at D5 (P < 0.01). In addition, miR-182 levels decreased significantly in the oviduct but increased significantly in the ovary at D15 compared with D5 (P < 0.01).

### The effect of E2/P4 on the expression levels of miR-182 in EEC

**Fig 3:**
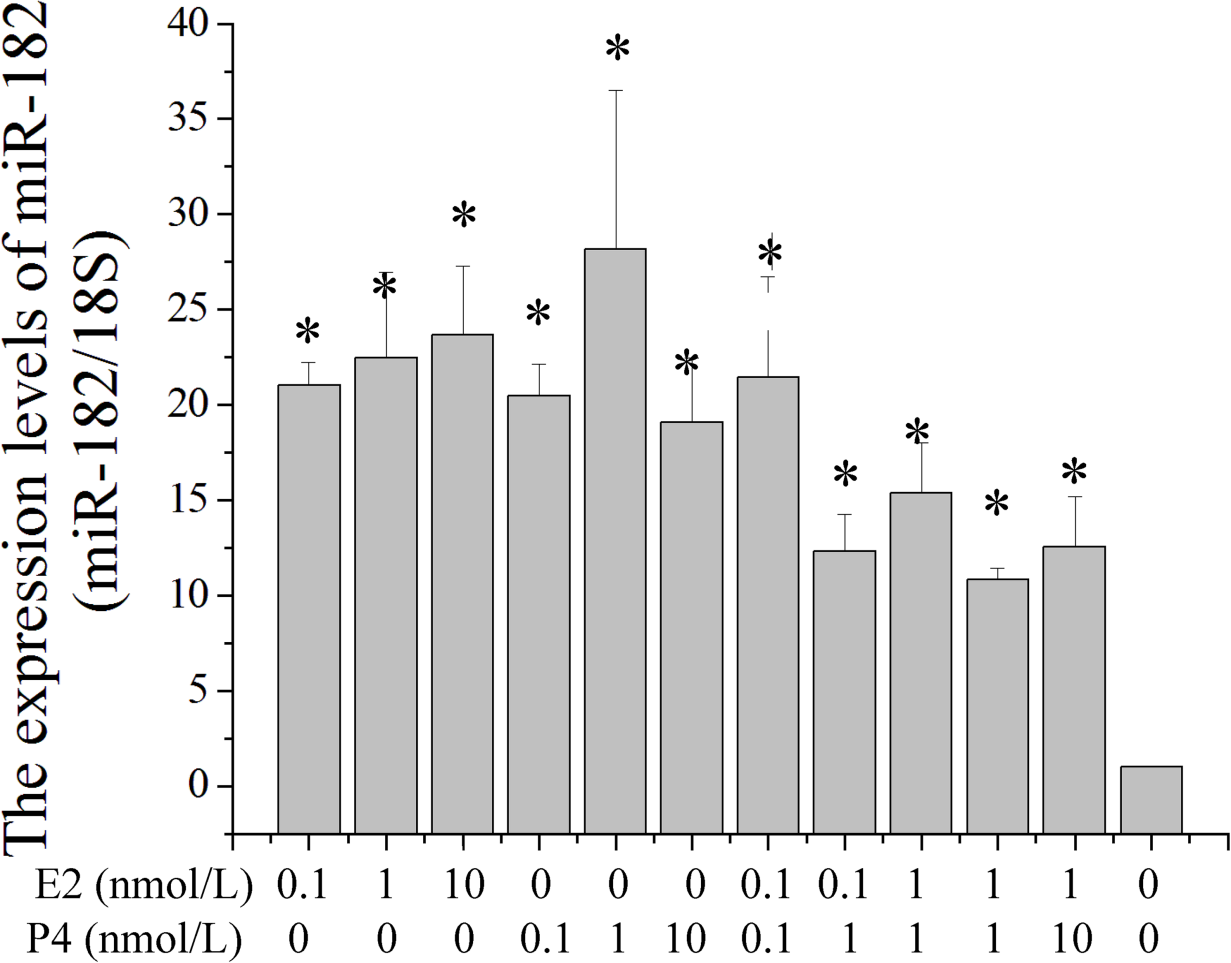
The effect of E2 and P4 on the expression levels of miR-182 in endometrial epithelium cells (EECs). The effect of E2 and P4 on the expression levels of miR-182 in EEC were measured by Stem-loop RT-qPCR and normalized to 18S. Values were showed as “means ± SD” (n = 3×3). * mean that the difference was significant compared to the control cells (did not added E2 and P4 in cell medium).

To investigate the response of miR-182 expression levels on sex hormones in EECs, β-estradiol (E2) and progesterone (P4) were diluted in cell medium to different concentrations. The expression levels of miR-182 were significantly enhanced with the constituents of E2, and the highest level was 10 nM in this study. The highest level of miR-182 was observed after treatment with 1 nM P4. The miR-182 levels were down-regulated when the cells were treated with combination of E2 and P4 (Fig 3). This result suggested that the expression levels of miR-182 were affected by sex hormones in EECs.

### miR-182 affected the surface microtopography of EEC

**Fig 4:**
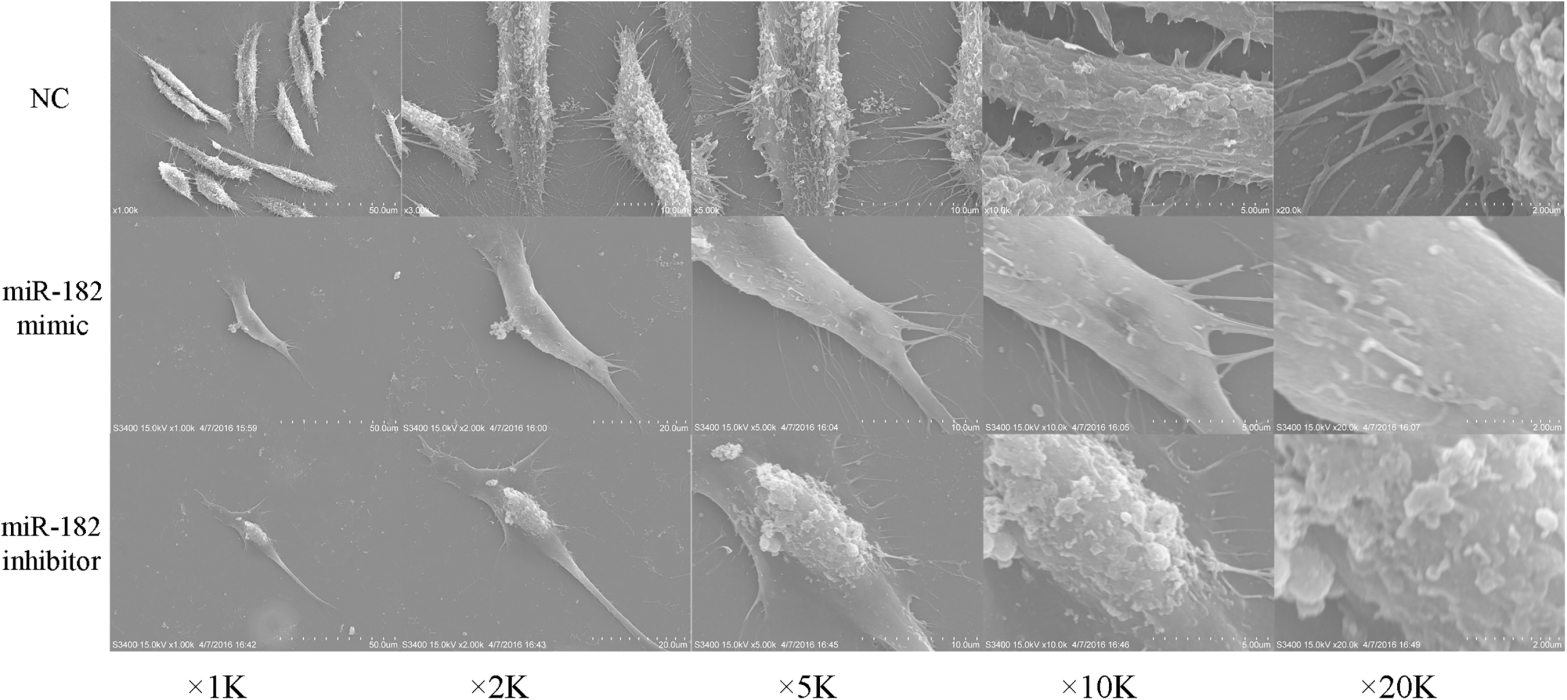
The effect of miR-182 on the surface microtopography of endometrial epithelium cells (EECs). The contours of miR-182 mimic-transfected EEC cells are clear and microvilli regularly distributed on the surface; after the EEC cells were transfected with miR-182 inhibitor, cells became significantly shrinked and gradually became round shape with poorly adhesion.

Prior to the receptive phase in the uterus, the surface epithelium is characterized by a dense distribution of long, thin microvilli that are covered with a uniformly glycocalyx, are under hormonal control and vary in length and number with the estrous cycle and during pregnancy [41]. On the surface cells treated with mimics of miR-182, microridges with uniform and regularly distributed microvilli were observed. However, cells treated with miR-182 inhibitors became significantly shrinked and gradually became round shape with poorly adhesion (Fig 4).

### miR-182 regulated cell proliferation, cycle and apoptosis of EEC

**Fig 5:**
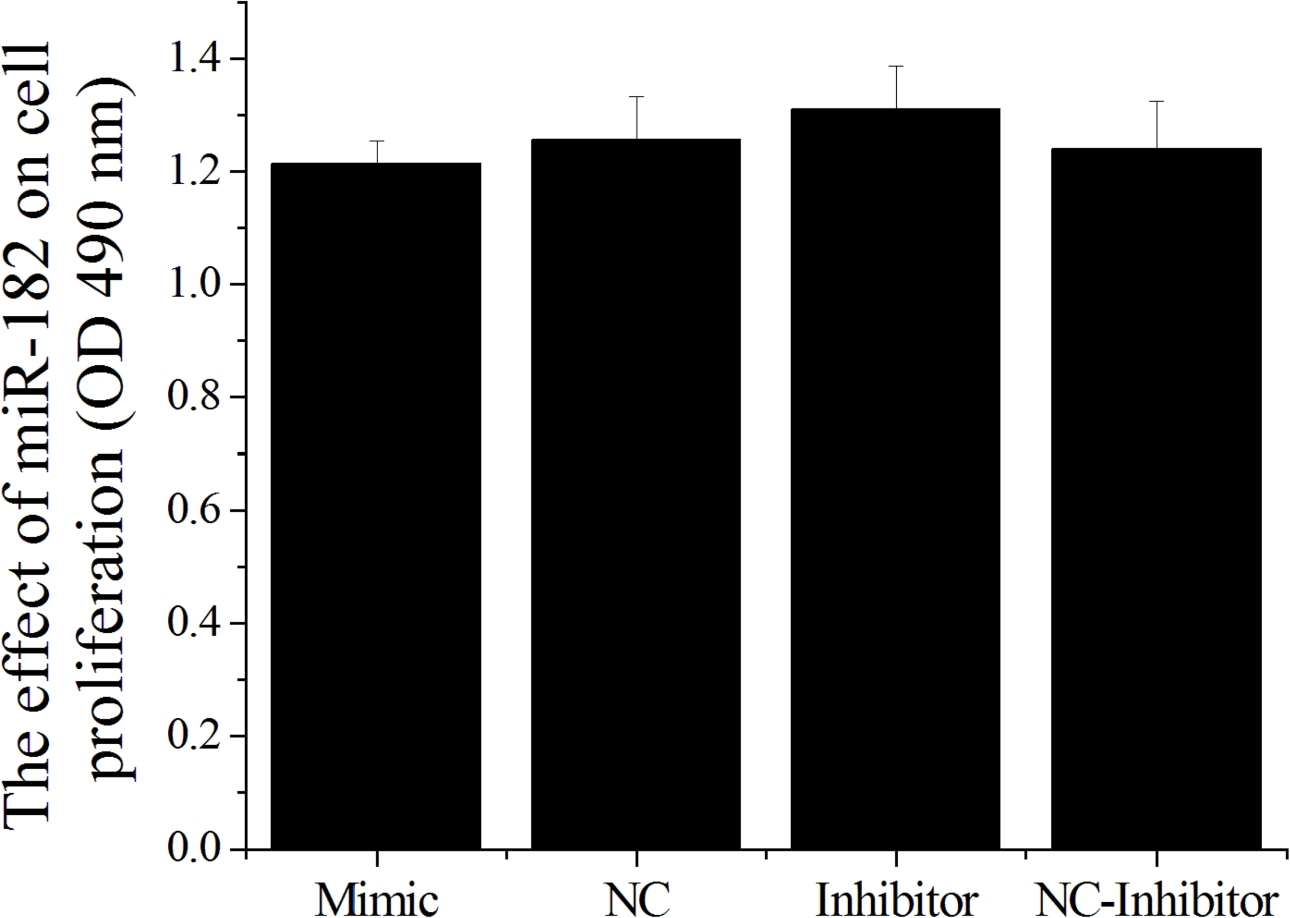
The effect of miR-182 on the proliferation of endometrial epithelium cells (EECs). The effect of miR-182 on the proliferation of EEC was measured by MTT. Values were showed as “means ± SD” (n = 3×7). ** indicates that P < 0.01 and * indicates that P < 0.05.

**Fig 6:**
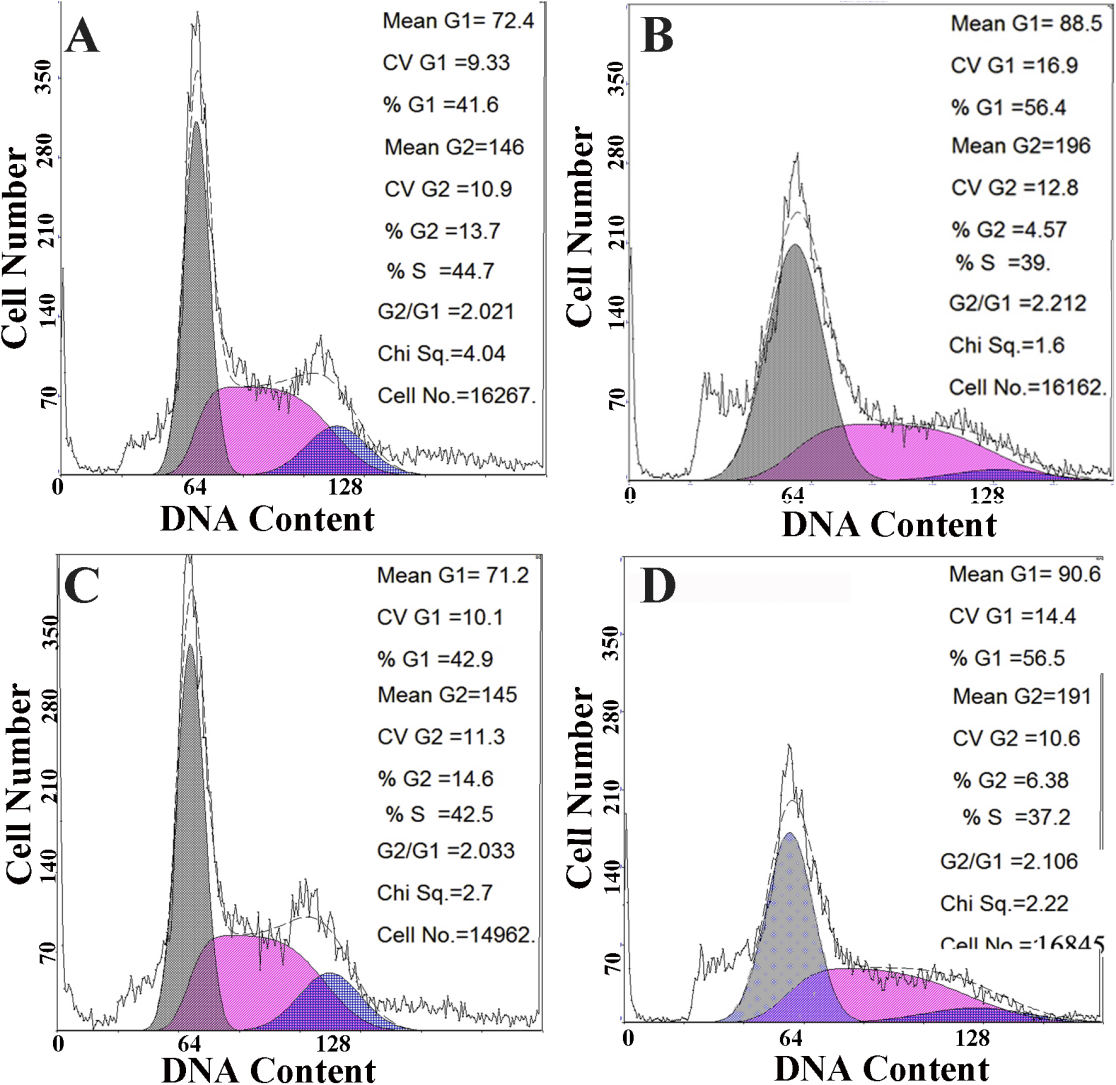
The effect of miR-182 on the cell cycle of endometrial epithelium cells (EECs). The cell cycle analysis of EEC were made after the cells were transfected with miR-182 mimic (A); NC (B); miR-182 inhibitor (C); NC inhibitor (D).

In this study, MTT assay (3-(4,5-dimethyl-2-thiazolyl)-2,5-diphenyl-2-H-tetrazolium bromide) showed that miR-182 mimics inhibited cell proliferation and miR-182 inhibitors promoted cell proliferation, but the differences were not statistically significant in EEC cells (Fig 5). Cell proliferation is generally regulated by progression through the cell cycle [42]. Consequently, disruption of the cell cycle is considered the common cause behind the inhibition of cell proliferation [43]. To determine whether the anti-proliferative effect of miR-182 is due to cell cycle disruption, flow cytometry was used to analyze the changes in the cell cycle in EECs. The DNA contents of miR-182-treated or control NC cells were quantitated for cell cycle analysis. The distribution of cells in the different phases of the cell cycle did not significantly change following miR-182 exposure in EECs (Fig 6), suggesting that miR-182 did not cause EEC cell cycle arrest under the conditions described.

**Fig 7:**
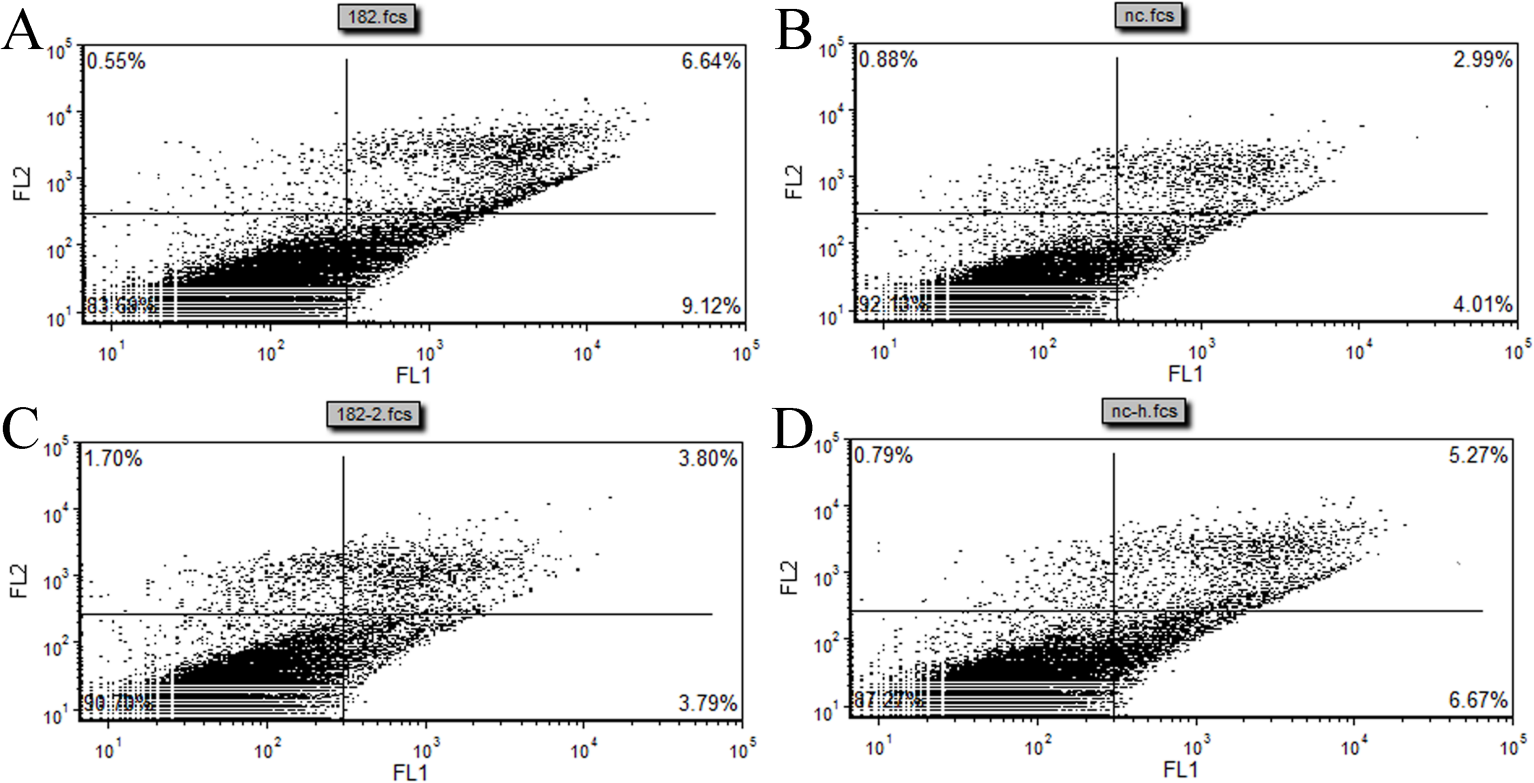
The effect of miR-182 on the cell apoptosis of endometrial epithelium cells (EECs). The cell apoptosis analysis of EEC were made after the cells were transfected with miR-182 mimic (A); NC (B); miR-182 inhibitor (C); NC inhibitor (D).

In addition, an Annexin V-FITC/PI assay combined with flow cytometry 48 h after transfection with miR-182 mimics or inhibitors, NC or NC- inhibitors showed increased apoptosis of EECs (Fig 7A–7D) upon treatment with the miR-182 mimics compared with NC. The apoptosis rate of EECs treated with the scrambled miR-182 mimics was 15.76%, and the apoptosis rate decreased to 7.59% when treated with the miR-182 inhibitors. This study suggested that miR-182 induced EECs apoptosis.

### PTN are differentially expressed in the pre-receptive and receptive endometrium

Constitutive expression of PTN in adults is limited to cells such as testicular Leydig cells, uterine cells, and discrete populations of glia and neurons [44–47]. Hormones, such as estrogen and testosterone, are regulators of PTN gene expression [48], and a further study has shown that PTN is an IFNT-regulated gene in the glandular epithelial cells [27].

**Fig 8:**
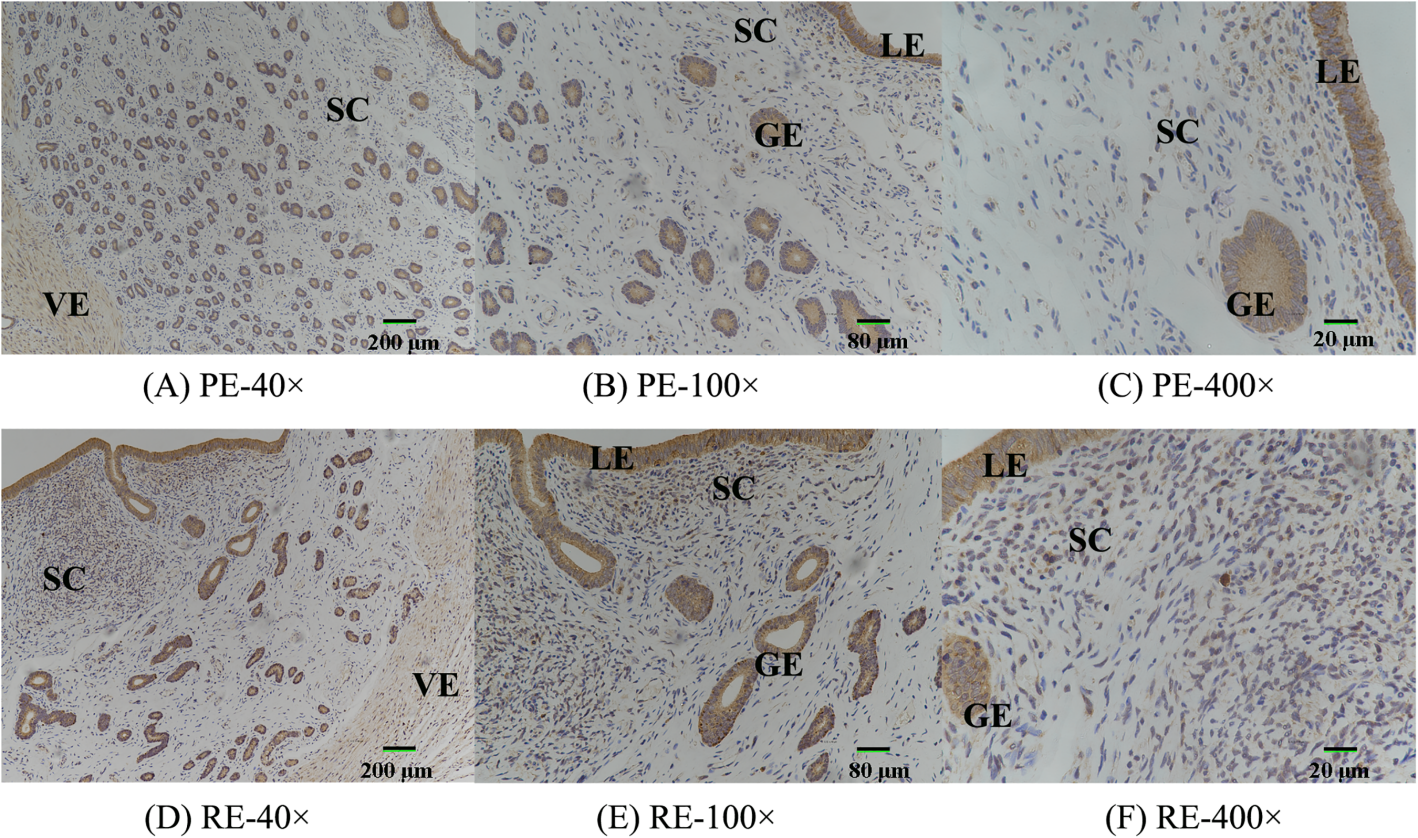
The expression levels of PTN protein in the endometrium at D5 and D15. The protein levels were detected by Immunohistochemical staining in the endometrium at D5 and D15. LE, luminal epithelium; GE, glandular epithelium; SC, stroma cell; VE, vascular endothelial cell. Original magnification ×40 (A, D, ruler marker = 200 μm), ×100 (B, E, ruler marker = 80 μm), ×400 (C, F, ruler marker = 20 μm).

In the previous study, we adopted the Illumina Solexa technology to obtain a large and reliable transcriptomic dataset from the PE (D5) and RE (D15) in dairy goats. A comprehensive analysis of the mRNA was constructed, and PTN was found to be decreased 20.97-fold in the RE compared with the PE. In the present study, we also observed that the luminal and glandular epithelia, which are vascular endothelial and smooth muscle cells in the endometrium, respectively, displayed a strong cytoplasmic distribution pattern for PTN (Fig 8), and this PTN immunoreaction intensity (DOI) was lower at D15 compared with D5 (Table 3).

**Table 3:** The immunohistochemical staining results of PTN in uterus of dairy goats.

Index (Mean)	100×		400×	
	D5	D15	D5	D15
**Area**	118.37±5.6	115.19±10.34	626.15±10.85**	155.83±27.63
**Density**	0.28±0.02	0.24±0.13	0.28±0.02	0.28±0.02
**IOD**	34.54±1.67	31.33±4.01	192.68±33.85**	46.15±8.60

Note: IOD was integrated optical density.

“**”, p< 0.01.

### miR-182 directly regulates PTN through its 3′ UTR

Human PTN was predicted to be the target of miR-182 based on the information from Targetscan (www.targetscan.org) and miRanda (www.microrna.org). Importantly, the predicted target site is also conserved, and miR-182 was found to directly target goat PTN through its 3′-UTR sequence. Therefore, we hypothesized that miR-182 down-regulates the expression of PTN in the endometrial cells of dairy goats.

**Fig 9:**
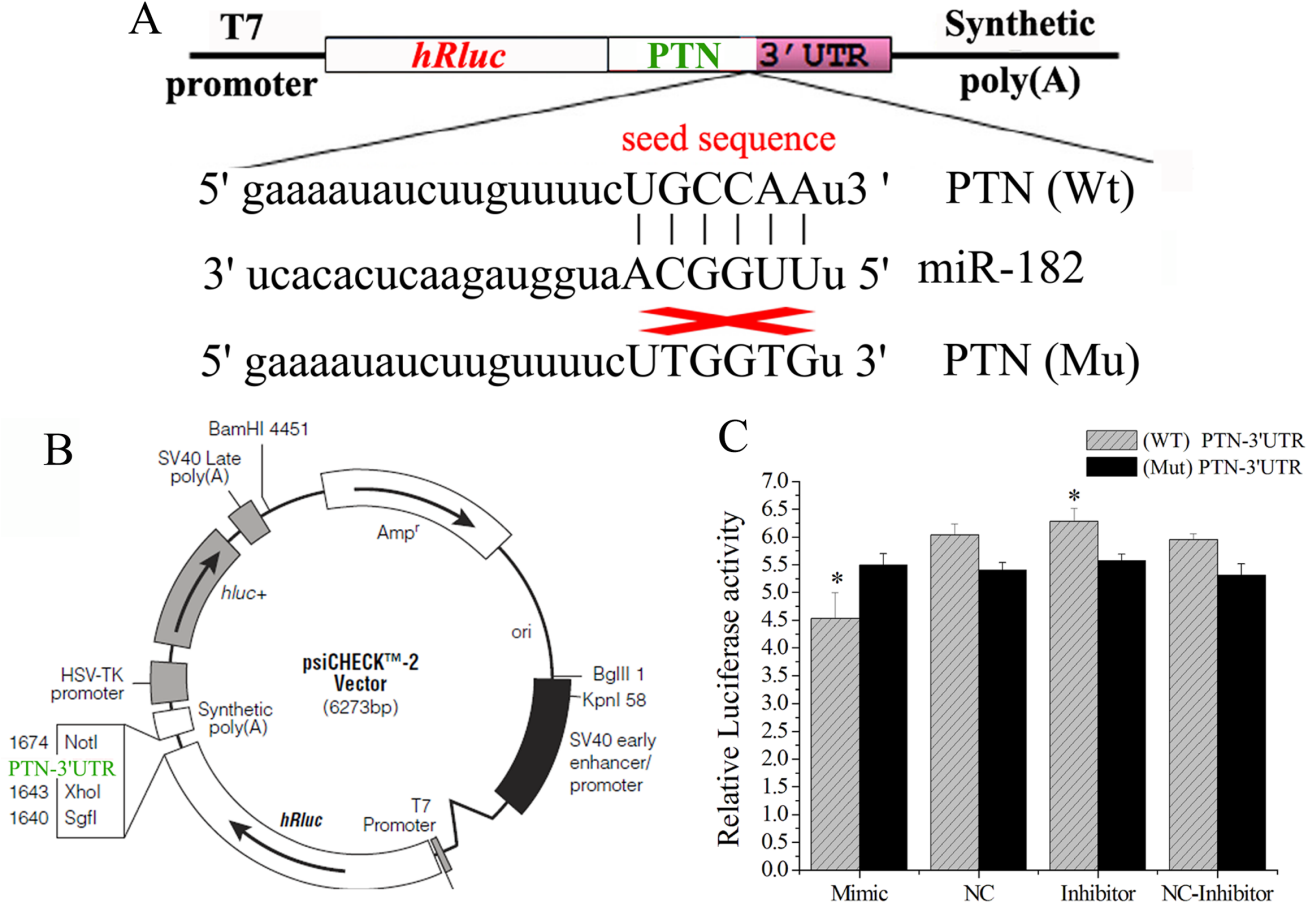
miR-182 down-regulated the expression level of PTN via the 3′ UTR. (A) Schematic diagram illustrating the design of luciferase reporters with the WT- PTN 3′ UTR (WT-PTN) or the site-directed mutant PTN 3′ UTR (Mut-PTN). The nucleotides in red represented “seed sequence” of miR-182, the mutation nucleotides were in yellow color. (B) psiCHECKTM-2 vector map and the insertion site of PTN-3′UTR marked in green color. (C) The PTN -3′ UTR or its mutation luciferase reporter vectors were co-transfected with miR-182 mimic (or negative control) into 293T cells. Luciferase assay was performed 24 h after transfection. Results are represented as relative luciferase activity. The results are represented as mean ± SD (n = 3×3); *p < 0.05, ** p < 0.01.

To determine whether miR-182 directly targets goat PTN through the predicted binding sites in the PTN 3′ UTR, the full-length 3′ UTR-containing miR-182 targeted sites were cloned and inserted downstream of the luciferase gene in the psiCHECKTM-2 reporter plasmid, and the mutated plasmids were constructed by inserting PTN-3′ UTR with the mutated miR-182 binding site (Fig 9A and 9B). The wild-type (psiCHECK-PTN-UTR-WT) or mutated (psiCHECK-PTN-UTR-Mut) plasmids were co-transfected with either the miR-182 mimics, miR-182 inhibitors, NC, or NC-inhibitors into HEK293T cells. After co-transfection with the plasmids, the luciferase activity of the miR-182 group was significantly lower than that of the NC group (P < 0.01) after 48 h after transfection, and this reduction was rescued in the mutation groups (Fig 9C). These results confirm that miR-182 targets goat PTN.

### miR-182 regulates PTN expression in EECs

**Fig 10:**
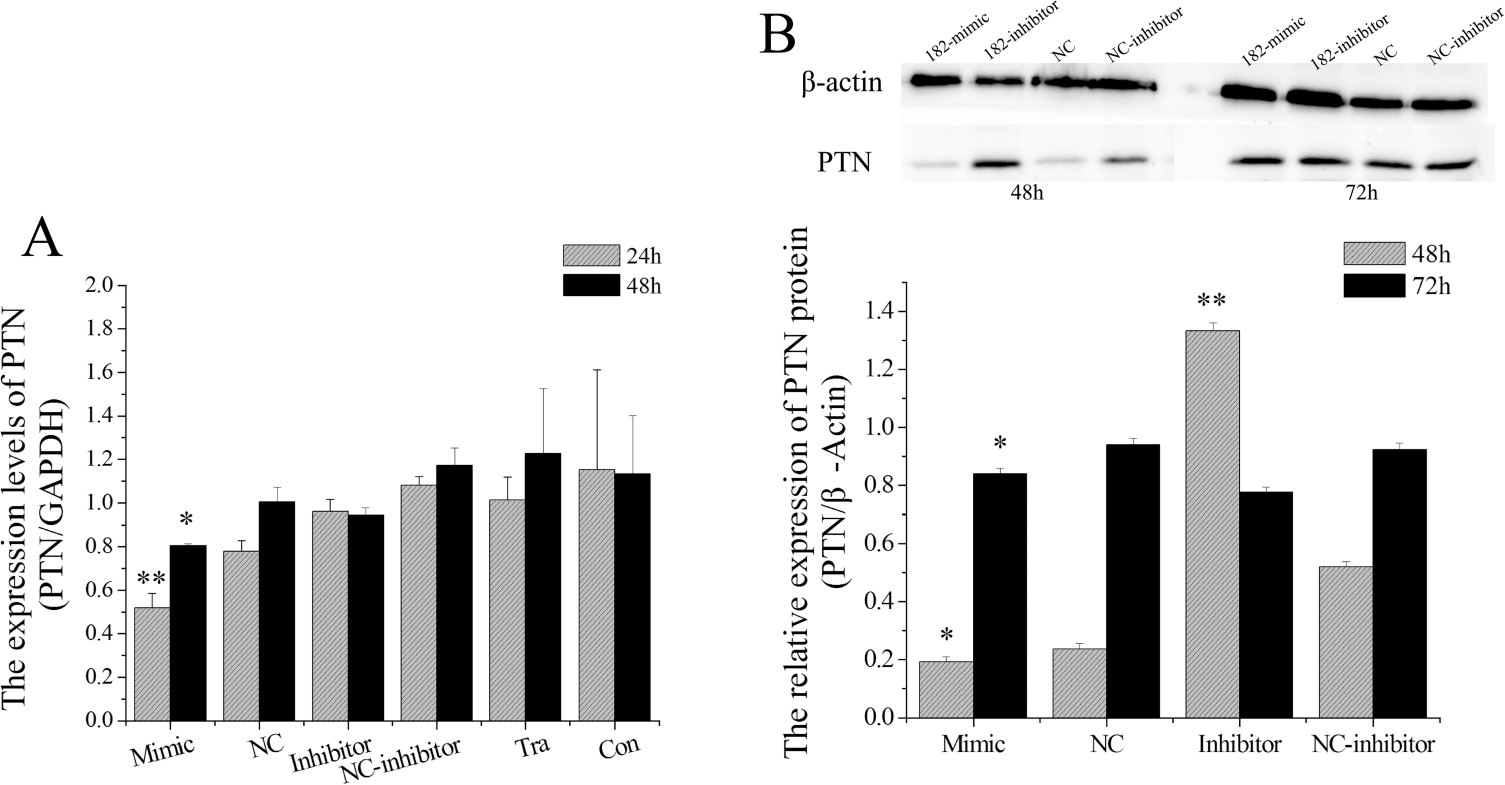
miR-182 down-regulated the PTN in endometrial epithelium cells (EECs). (A) miR-182 down-regulated the PTN mRNA levels in EEC, PTN mRNA levels were measured by RT-qPCR and normalized to GAPDH. Values were showed as “means ± SD”, ** indicates that P < 0.01 and * indicates that P < 0.05. (B) PTN protein levels were measured by WB, PTN densitometry was normalized to the β-actin density from the same lane. Each experiment was repeated three times in triplicate, the results are represented as mean ± SD (n = 3×3); *p < 0.05. The date was analyzed only compared to its corresponding NC at the same timing.

After confirming the high abundance of miR-182 and low levels of PTN mRNA in the RE of goats, we further investigated if miR-182 down-regulates the expression levels of PTN in EECs. We transfected EECs with a miR-182 mimics, miR-182 inhibitors, NC (negative control), or NC-inhibitors at a cell density of 80–90% using Lipofectamine 2000. The mRNA levels of PTN significantly decreased at both 24 h (P < 0.01) and 48 h (P < 0.05) after the EEC was transfected with the miR-182 mimics (Fig 10A). However, the miR-182 inhibitors had no effect on the mRNA expression levels at 24 h and 48 h (P > 0.05).

We further quantified PTN protein expression by performing WB analysis. PTN protein levels were significantly decreased in miR-182-transfected cells compared with NC at both 48 h and 72 h (P < 0.05), and miR-182 inhibitors significantly increased PTN levels in EECs at 48 h (P < 0.01) but not at 72 h (P > 0.05).

### miR-182 regulates AKT expression in EECs

**Fig 11:**
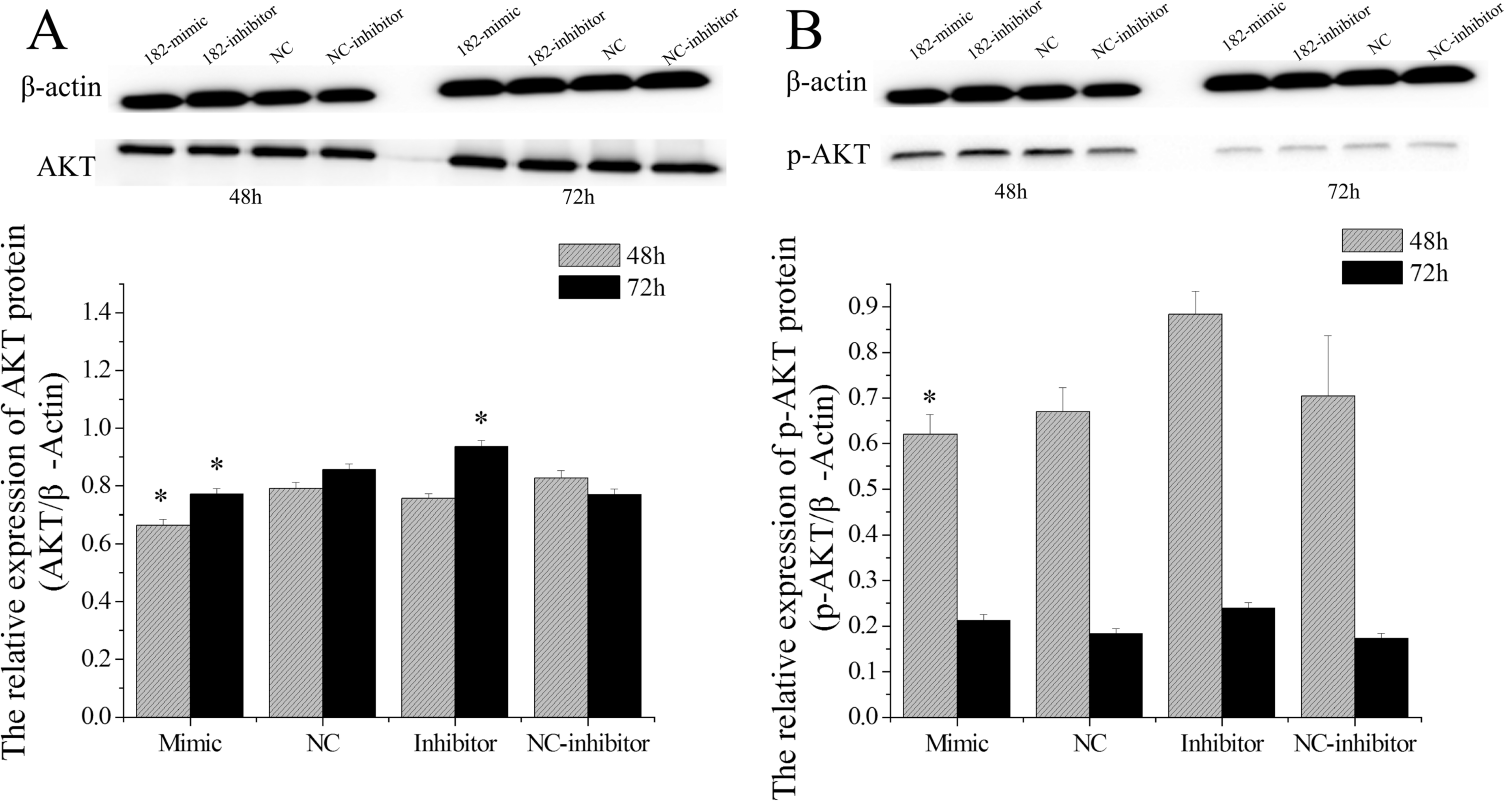
The effect of miR-182 on AKT and p-AKT levels in endometrial epithelium cells (EECs). AKT protein levels (A) in EEC cells, p-AKT protein levels (B) were measured by WB, densitometry was normalized to the β-actin density from the same lane. Each experiment was repeated three times in triplicate, the results are represented as “means ± SD” (n = 3×3), ** indicates that P < 0.01 and * p < 0.05. The date was analyzed only compared to its corresponding NC at the same timing.

AKT is the central node in the PTEN-regulated pathway, and activated AKT has been shown to promote cell cycle progression, cell growth, cell survival, cell migration, angiogenesis, protein synthesis, and glucose metabolism through phosphorylation of its substrates [49]. A recent study suggested that PTN plays a critical positive feedback role in controlling AKT activity [50]. The above results suggest that miR-182 regulates PTN expression in EECs, and we sought to analyze AKT protein levels after the cells were treated with miR-182. We found that miR-182 decreased the expression of total AKT protein levels both at 48 h (P < 0.05) and 72 h (P < 0.05), and the miR-182 inhibitors increased AKT levels at 72 h (P < 0.05, Fig 11A). Furthermore, miR-182 also decreased p-AKT protein levels at 48 h (P < 0.05), and the miR-182 inhibitors increased p-AKT levels at both 48 h (P < 0.05) and 72 h (P < 0.05, Fig 11B).

### miR-182 regulates the expression of PTN-related genes

As a growth factor, PTN stimulates the mitogenesis of fibroblasts, endothelial cells, and epithelial cells in culture, as well as neurite outgrowth in neonatal neuronal cells [51, 52]. Homeostasis of endometrial tissue plays an important role in the preparation of the uterus for each new estrous cycle or to support pregnancy [41]. Previous studies have evaluated cell proliferation and apoptosis in the endometrium during the estrous cycle. Apoptosis of endometrial cells has been studied in sow [53], canine [54, 55], rabbit [56] and women [57]. However, to our knowledge, no such study has yet been undertaken in the dairy goat. Thus, the protein levels of Bcl-2, FAS, MAPK, Caspase-3, and SP1 were detected in the EECs treated with miR-182.

**Fig 12:**
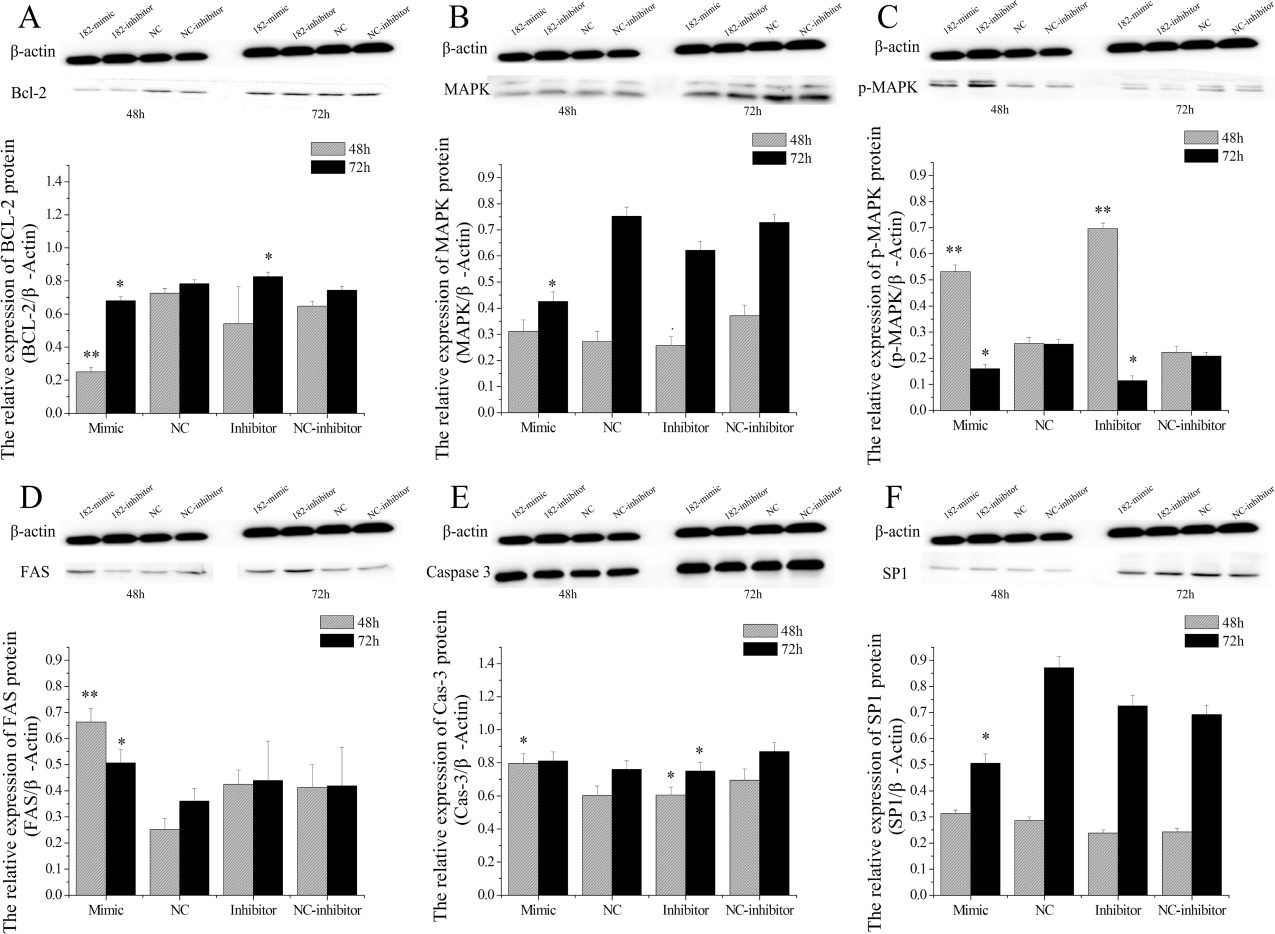
The effect of miR-182 on PTN-related protein levels in endometrial epithelium cells (EECs). Bcl-2 (A), MAPK (B), p-MAPK (C), FAS (D), Caspase-3 (E), and SP1 (F) protein levels were measured by WB, densitometry was normalized to the β-actin density from the same lane. Each experiment was repeated three times in triplicate, the results are represented as “means ± SD” (n = 3×3), ** indicates that P < 0.01 and * p < 0.05. The date was analyzed only compared to its corresponding NC at the same timing.

Previous studies have confirmed that the Bcl-2 family proteins are regulated during the involution of endometrium cells. Thus, we proposed that the expression of Bcl-2 family proteins may be altered under miR-182 conditions in EECs. Bcl-2 protein expression levels were decreased significantly 48 h after treatment with miR-182 mimics (P < 0.01) and 72 h (P < 0.05), and the levels were significantly up-regulated after the EECs were treated with the miR-182 inhibitors at 72 h (P < 0.05). Confusingly, miR-182 inhibitors down-regulated the protein expression level of Bcl-2 at 48 h (P < 0.01) (Fig 12A).

Furthermore, the expression of proteins upstream of Bcl-2, MAPK (p44/42) and p-MAPK (p44/42), were quantified by WB analyses. The expression of total MAPK and p-MAPK proteins were detected, and these data showed that miR-182 decreased the MAPK protein levels in EECs at 72 h (Fig 12B). Surprisingly, both the miR-182 mimics and inhibitors increased the p-MAPK levels at 48 h (P < 0.01) and decreased at 72 h (P < 0.05, Fig 12C). The miR-182 mimics increased the protein expression levels of FAS in EECs at 48 h (P < 0.01) and 72 h (P < 0.05) (Fig 12D). The expression levels of FAS were not affected in miR-182 inhibitors-treated EECs at 48 h (P < 0.01).

The morphological alterations observed in different cells undergoing apoptosis were due to the activation of specific enzymes known as “Caspases”. The activation of these enzymes modulate apoptosis, and they serve as markers of apoptosis before morphological signs are evident [41]. Caspase-3 is a key protease involved in the execution of apoptosis. The miR-182 mimics increased the Caspase-3 protein levels at both 48 h (P < 0.05) and 72 h (P > 0.05) in EECs (Fig 12E).The miR-182 inhibitors decreased Caspase-3 levels at 48 h (P < 0.05) and 72 h (P < 0.05). Furthermore, the miR-182 mimics decreased SP1 protein levels at 48 h (P < 0.05) in EECs (Fig 12F), but the difference was not significant at 72 h (P > 0.05). In addition, miR-182 inhibitors did not affect SP1 protein levels at either the 48 h or 72 h time points in EECs.

### miR-182 regulates the expression of endometrial receptivity marker genes

Endometrial receptivity is the result of the synchronized and integrated interaction between ovarian hormones, growth and transcription factors, lipid mediators, and cytokines with paracrine signaling [58], and rebuilds the phenotype that allows embryo adhesion and placentation to occur [59]. More recently, several morphological and biochemical endometrial receptivity biomarkers were proposed, including OPN [60], VEGF [61], COX-2 [62] and PRLR [63, 64] during the WOI [65]. Therefore, changes in OPN, VEGF, COX-2 and PRLR protein levels were investigated in EECs after they were treated with miR-182 mimics, miR-182 inhibitors, NC (negative control), or NC-inhibitors.

**Fig 13:**
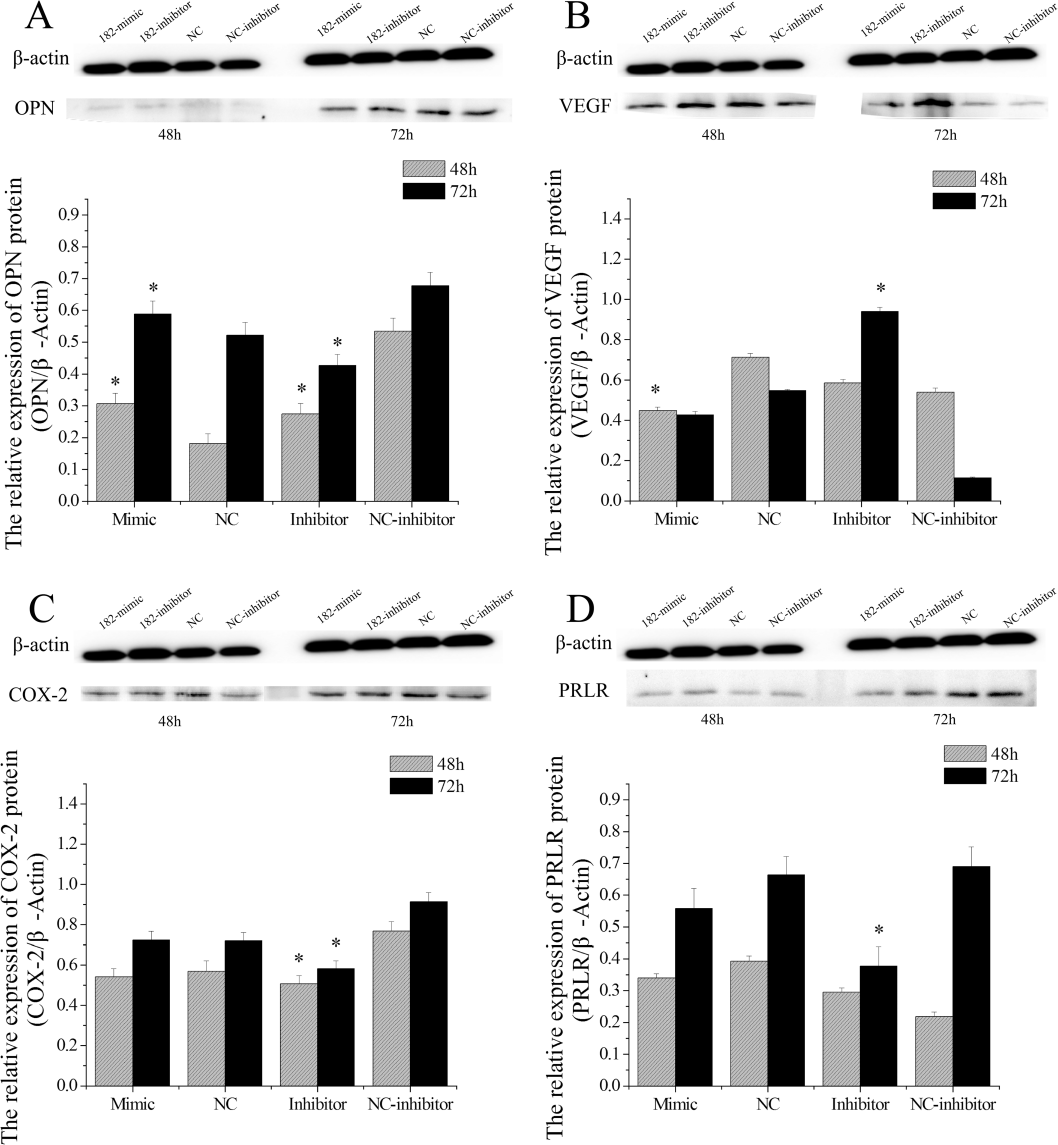
The effect of miR-182 on some marker protein of endometrial receptivity in endometrial epithelium cells (EECs). The protein levels of OPN (A), VEGF (B), COX-2 (C) and PRLR (D) were measured by WB, densitometry was normalized to the β-actin density from the same lane. Each experiment was repeated three times in triplicate, the results are represented as “means ± SD” (n = 3×3), ** indicates that P < 0.01 and * p < 0.05. The date was analyzed only compared to its corresponding NC at the same timing.

The OPN protein levels were up-regulated at both 48 h and 72 h (P < 0.05) in miR-182 mimics-treated EECs. In addition, miR-182 inhibitors decreased OPN levels at 48 h and 72 h (P < 0.05, Fig 13A). The miR-182 mimics decreased VEGF protein levels in EECs at 48 h (P < 0.05) but not at 72 h (P > 0.05), and the miR-182 inhibitors increased VEGF protein levels at 72 h but not 48 h (Fig 13B), suggesting that the miR-182 mimics and inhibitors exert their effects on different timelines.

No statistical differences were found in COX-2 levels between the miR-182 mimics and NC in EECs at either 48 h or 72 h (P > 0.05), but COX-2 expression levels in the EECs were significantly decreased 48 h and 72 h after treatment with the miR-182 inhibitors compared with NC (P < 0.05). Furthermore, the miR-182 inhibitors decreased PRLR protein levels at 72 h (P < 0.05) in EECs (Fig 13D), but no significant difference in PRLR protein level was found with the mimics (P > 0.05).

## Discussion

In recent years, more and more miRNAs have been recently identified and play important roles in post-transcriptional regulation of gene expression in the endometrium, such as miR-183 regulate ITGB1P expression and promote invasion of ESC cells [66]. Functional studies indicated that miR-183 might contribute to human endometrial stromal cell apoptosis and impose a negative regulatory impact on cell invasiveness in humans [67]. In addition, miR-21 is highly expressed in the sub-luminal ESC cells at implantation sites is regulated in active blastocysts in the mouse [68].

### The expression of miR-182 in dairy goats

miR-182 was first identified as a retina-specific miRNA, with its expression abundantly increasing postnatally and reaching the peak in adult retina [14]. Further study showed that miR-182 displayed a diurnal variation in the retina of mice, revealed its role in retina development and the regulation of mammalian circadian rhythm [69]. What’s more, miR-182 is demonstrated to function either as a tumor suppressor or an oncomir in various human cancers [12, 17, 70]. Our previous sequencing data have indicated that miR-182 is a differentially expressed miRNA with a 15.55-fold increase in the RE compared with the PE of dairy goats [30]. In the present study, the expression level of miR-182 in the PE was remarkably higher than that in the RE (Fig 2), which was inconsistent with the previous sequencing data [30]. Furthermore, this study showed extensive expression of miR-182 in other tissues, especially in the kidney at D5 and oviduct at D15.

### The effect of E2/P4 on the expression levels of miR-182 in EECs

E2 and P4 play leading roles in the endometrium of animals, and EECs form the first cell-layer that has physical and physiological contact with the blastocyst trophectoderm [71]. Under the coordination of E2 and P4, EECs undergo structural and functional changes that establish uterine receptivity [72], which is absolutely necessary for successful embryo implantation in all animals.

To investigate the response of miR-182 expression levels to sex hormones in EECs, E2 and P4 were diluted in cell medium to different concentrations. The expression levels of miR-182 were significantly enhanced in a concentration-dependent manner, with the highest level observed in the presence of P4 alone with 1 nM. This study is the first to our knowledge that the expression levels of miR-182 are affected by sex hormones in the EECs of dairy goats. Thus, the induction of miR-182 may be a critical event for the development of endometrial receptivity, and this induction may be regulated by E2 and P4.

### The effect of miR-182 on the surface morphology of EECs

Changes of the luminal epithelial cell surface to facilitate adhesive properties are critical for the attainment of receptivity and successful embryo adhesion [71, 73, 74]. Prior to the receptive phase in the rat uterus, the surface epithelium is characterized by a dense distribution of long, thin microvilli that are covered with a uniform glycocalyx and that are under hormonal control and vary in length and number during the estrous cycle and pregnancy [41]. Based on the above information, the EEC surface morphology was observed by scanning electron microscopy after the cells were transfected with a miR-182 mimics or inhibitors in this study. On the surface of cells treated with miR-182, we observed microridges with uniform and regularly-distributed microvilli. However, cells treated with a miR-182 inhibitors became round, the microvilli on the surface of the cell membrane disappeared, and the cell membrane sprouted (buds) and formed apoptotic bodies (Fig 4). This result suggested that miR-182 may be indispensable for the establishment of endometrial receptivity in dairy goats.

### miR-182 regulates proliferation, cell cycle, and apoptosis in EECs

A critical event during embryo implantation is the extensive tissue remodeling at the maternal-fetal interface, characterized by cell proliferation, cell migration and cell adhesion [75, 76]. Integrins, extracellular matrix (ECM) and matrix metalloproteinases (MMPs) are involved in this process and are regulated by the complex endocrine, autocrine and paracrine milieu within the uterus and during cancer [77], and angiogenesis is a common feature of both implantation and cancer spread [78, 79]. Moreover, it is known that miR-182 up-regulated in various cancer type [16, 80, 81], striking similarities are present between the behavior of placental cells during the WOI and that of cancer cells [82]. Nevertheless, the transcriptional regulation of miR-182 as well as its role in EECs during WOI is not clear.

The MTT assay has been widely used to measure the viability and proliferation of cells. In his study, an MTT assay showed that the miR-182 mimics inhibited and miR-182 inhibitors promoted the proliferation of EECs (Fig 5), although the differences were not significant (P > 0.05). Cell proliferation is generally regulated by progression through the cell cycle [42]. Consequently, the disruption of the cell cycle is considered to be a common cause of cell proliferation inhibition [43]. To determine whether the anti-proliferative effect of miR-182 was due to cell cycle disruption, flow cytometry was used to analyze changes in the cell cycle. The distribution of cells in the different phases of the cell cycle did not significantly change after miR-182 exposure in EECs (Fig 6), suggesting that miR-182 does not cause EEC cell cycle arrest under the conditions described.

In addition, an Annexin V-FITC/PI assay combined with flow cytometry 48 h after transfection with miR-182 mimics, miR-182 inhibitors, NC or NC-inhibitors showed that EEC apoptosis (Fig 7) increased upon the introduction of miR-182 mimics compared with NC. The apoptosis rate of EECs treated with a scrambled miR-182 mimics was 15.76%, and this rate decreased to 7.58% upon treatment with miR-182 inhibitors, suggesting that miR-182 induces EEC apoptosis in this study.

### miR-182 regulates PTN expression in EECs

Many experiments have confirmed that miRNAs play diverse biological functions by targeting and down-regulating protein levels in animals. For example, Let-7g was reported to target caspase-3 and inhibit the apoptosis induced by oxidized low density lipoprotein (OX-LDL) in endothelial cells [83]. MiRNA-29b promotes high-fat diet-stimulated endothelial permeability and apoptosis in apoE knock-out mice by down-regulating MT1 expression [84], miR-21 is overexpressed in response to high glucose and protects endothelial cells from apoptosis by targeting death-domain associated protein [85].

We confirmed the high abundance of miR-182 and the lower levels of PTN in the receptive endometrium of goats and miR-182 directly regulates PTN through its 3′UTR. We further investigated whether miR-182 down-regulated the expression levels of PTN in EECs of dairy goats. And the result showed that overexpression of miR-182 dramatically decreased the mRNA levels of PTN at 48 h; in contrast, miR-182 inhibition increased PTN levels in EECs. WB results further indicated that PTN was down-regulated by miR-182 in dairy goats at 48 h, however, inhibition of PTN protein expression by miR-182 disappeared at 72 h. And the reasoning behind that would be miR-182 mimics used in this study was an artificially synthesized mature miRNA with a shorter effective time; thus, its function may have reduced with time.

### miR-182 regulates the phosphorylation of AKT

As a growth factor, PTN could stimulate the cells mitogenesis in culture is dependent on tyrosine kinase activation and utilizes the mitogen-activated protein kinase and the PI3K pathways to transduce a mitogenic signal [51, 86]. Further studies show that PTN could induce the activation of the PI3K/AKT-related pathway in bovine epithelial lens cells [51], glioblastoma cells [87], NIH3T3 fibroblasts [88], and human umbilical vein endothelial cells [89]. Phosphorylated AKT (activated) modulates the activity of a variety of downstream proteins that are related to cell growth, proliferation, apoptosis or differentiation [90, 91]. In addition, inhibiting the PI3K/Akt pathway reversed progestin resistance in endometrial cancer [92]. In the present study, miR-182 decreased the expression of total AKT protein levels at both the 48 h and 72 h time points, and the miR-182 inhibitors increased the levels at 72 h (Fig 11A). Meanwhile, miR-182 decreased the p-AKT protein levels at 48 h, and the miR-182 inhibitors increased the levels at both 48 h and 72 h (Fig 11B). In short, these results were in accordance with previous study that reported that knocking down the expression of PTN by small interfering RNA resulted in the reduction of AKT [50].

### miR-182 affects the expression of apoptosis-related genes

In mammals, endometrial cells are remodeled by apoptosis, proliferation and differentiation throughout the estrous cycle [93], and apoptosis is an important process in the reconstruction of endometrial receptivity and early implantation of the embryo [94]. An imbalance of pro- and anti-apoptotic events in the secretory endometrium seems to be involved in implantation disorders and consecutive pregnancy complications [95]. What’s more, locally regulated apoptosis of EECs induced by the embryo is a crucial step to facilitate embryo anchorage and access to maternal blood supply in rodents [96] and human [19].

Both the mitochondrial pathway (Bcl-2 family) and the death-receptor system (Fas/FasL) involved in the apoptosis of the endometrium in humans [57, 97, 98] and monkeys [99]. However, no such study has been performed in the EECs of dairy goats. The present report for the first time studies the effect of miR-182 on apoptosis-related proteins (Bcl-2, FAS, and Caspase-3) in the EECs of dairy goats. Bcl-2 family proteins are critical regulators of programmed cell death and act by permeabilizing the mitochondrial outer membrane [100–102], and members of the Bcl-2 family can promote or inhibit apoptosis by synthesizing anti-apoptotic (i.e., Bcl-2, Bcl-Xl) or pro-apoptotic (i.e., BAX, BAK, BAD, BID, Bcl-Xs) proteins [103, 104]. In the present study, we found lower levels of BCL-2 protein in the miR-182-treated EECs, suggesting that miR-182 might induce EEC apoptosis by decreasing Bcl-2 expression. Furthermore, the upstream proteins of Bcl-2, MAPK (p44/42) and p-MAPK (p44/42), were also monitored by WB, and these results showed that miR-182 decreased the total MAPK levels at 72 h. Because of their key role in cell signalling, a rigorous regulation of MAPKs is essential in eukaryotic physiology [105], p-MAPKs target different downstream effectors that lead to changes in transcriptional programs [106].

Previous researchers believed there was a relationship of expression between anti- (Bcl2) and pro-apoptotic proteins (Fas, FasL, Bax), although the precise mechanism has not been fully clarified in canine endometrium [107]. Fas (also known as APO-1 or CD95), a tumor necrosis factor (TNF) superfamily receptor, is known to induce apoptotic cell death when it binds to FAS ligand (FasL) [108]. And FAS-mediated apoptosis occurs in the regulation of human endometrial function under hormonal regulation [109]. Further study showed that Fas was localized at the apical cell surface of hEEC, and flow cytometry revealed that 60% of hEECs express Fas [19]. Notably, the expression levels of FAS increased in miR-182 treated EECs at protein levels in dairy goats.

Caspase-3 (also termed CPP32, or apopain) is activated during the early stages of apoptosis [110] and it is the primary executioner of apoptosis, so the expression levels of Caspase-3 is one of the most common used detect indicator to evaluate the apoptosis of many cell types in previous studies [111, 112]. Furthermore, several studies have suggested that endometrial cell death is regulated by Caspase-3 during the menstrual or estrous cycle [113] and during early pregnancy [114]. In this study, we found that the miR-182 mimics increased the Caspase-3 protein levels at 48 h, and miR-182 inhibitors decreased the levels of Caspase-3.

Furthermore, Schulte reported a retroviral SP1 binding site in the PTN promoter is important for the expression in human choriocarcinoma cells [115]. SP1 is a ubiquitously expressed zinc finger protein that regulates the expression of genes by binding to a classical GC box (GGGCGGG),and its binding sites are located distal and/or proximal to the transcription start site of genes [116, 117]. Furthermore, SP1 can also interact with other proteins, such as BPV enhancer E2 protein [118], NF-kB [119], Rb [120], or E2F-1 [121] to contribute to tissue-specific gene regulation. In this study, EECs treated with the miR-182 mimics exhibited an obvious decrease in SP1 expression after 72 h. This result suggested that miR-182 may regulate SP1, but the specific molecular regulation mechanism needs further study.

### miR-182 may participate in the formation of endometrial receptivity

OPN has an arginine–glycine–aspartic acid (RGD)-binding site that can bind to the transiently expressed αvβ3 and α4β1 integrin heterodimers [122]. Further study suggested that OPN plays an important role in endometrial receptivity for its consistent up-regulation during the WOI [123]. Thus, we detected the protein levels of OPN in EECs and found that miR-182 significantly increased OPN protein levels in EECs, and the levels decreased after treatment with the miR-182 inhibitors.

In addition, VEGF express in the mouse and rabbit endometrium and participates in the increased angiogenesis and vascular permeability what are necessary for implantation [124, 125]. Deficient expression of VEGF and/or its receptors in mice result in poor development of the vascular network in the endometrium, leading to implantation failures and abortions [126]. Evidence suggests that the expression of VEGF is highly regulated in a temporal and spatial manner at the early stage of implantation [127]. In dairy goats, the VEGF protein levels were down-regulated by the miR-182 mimics, and the miR-182 inhibitors increased VEGF levels in the EECs.

In humans, P4 stimulates decidualization, which first begins in the endometrial stromal cells surrounding the spiral arteries of the uterus during the late secretory phase of the menstrual cycle [128]. At this time, the endometrium begins to undergo remodeling in preparation for embryo implantation. Specifically, the endometrial stromal cells (ESC) undergo a marked rearrangement of the intracellular architecture and begin to accumulate glycogen, initiating the secretion of various proteins, growth factors and cytokines [63], including prolactin (PRL). PRL is one of the strongest decidualization markers [129] involved in regulating endometrial secretory activity in human and rodent, but not in canine [130]. However, it is noteworthy that PRL cannot play important role in the signal transduction cascade without its receptor, PRLR [131]. In this study, the miR-182 mimics did not regulate the protein levels of PRLR in the EECs of dairy goats, but the miR-182 inhibitors down-regulated the levels, suggesting that miR-182 may be an essential factor to keep the expression of PRLR protein levels in the EECs of dairy goats.

Prostaglandin G/H synthase 2 (PTGS2 or COX2) is a rate-limiting enzyme for the synthesis of prostaglandin (PG), which is a critical factor for the maintenances of the uterine environment during early pregnancy [62]. In rodent uterus, COX2-derived PGE2 and PGI2 are the major mediators promoting vascular permeability and decidualization at implantation sites [132]. COX2 knockout mice are infertile, with abnormalities in ovulation, fertilization, implantation, and decidualization [133], and the anti-implantation effect of specific COX2 inhibitors results in decidualization defects [134]. We found that the COX2 protein levels were down-regulated by the miR-182 inhibitors, but the levels changed when the cells were treated with the miR-182 mimics, suggesting that miR-182 may be an essential factor to maintain the expression of COX2 protein levels in dairy goat EECs.

## Conclusion

Overall, this study has shown that miR-182 is widely expressed in different tissues in dairy goats and that the expression levels are regulated by E2 and P4 in EECs. We verified for the first time that miR-182 induced EEC apoptosis by directly targeting the 3’ untranslated region of PTN. In addition, miR-182 may be an essential factor for the regulation of the expression of OPN, COX2 and PRLR protein levels in dairy goat EECs. Thus, we conclude the following: miR-182 is up-regulated in the EECs during the WOI, and it induces cell apoptosis by down-regulating the expression of PTN at both mRNA and protein levels; miR-182 plays a role in the development of endometrial receptivity by down-regulating PTN levels and up-regulating or maintaining the expression levels of OPN, COX-2 and PRLR in dairy goat EECs.
